# Added value of integrative medicine to the practice of nursing in hospitals: results from a qualitative study in pediatrics

**DOI:** 10.1186/s12906-026-05533-w

**Published:** 2026-08-19

**Authors:** Alicia Reuse, Julie Dubois, Pierre-Yves Rodondi, Benedikt M. Huber

**Affiliations:** 1 Faculty of Science and Medicine, University of Fribourg, Fribourg, Switzerland; 2 Institute of Family Medicine, Faculty of Science and Medicine, University of Fribourg, Fribourg, Switzerland; 3 Center for Integrative Pediatrics, Department of Pediatrics, Fribourg Cantonal Hospital, Fribourg, Switzerland; 4 Department of Community Health, Faculty of Science and Medicine, University of Fribourg, Fribourg, Switzerland

**Keywords:** Nursing, Pediatrics, Integrative medicine, Integrative nursing, Complementary therapies, Qualitative research

## Abstract

**Background:**

Integrative medicine (IM) is increasingly being implemented in hospitals. In this context, nurses play a central role in the delivery of care and treatments. However, little is known about the influence of IM on the nursing profession. This is of particular interest in the current crisis of the nursing workforce, as previous studies have suggested that IM could be a way to address some of the issues linked to this crisis. The aim of this study was thus to explore nurses’ perceptions and experiences of practising IM in a pediatric hospital, as well as to identify barriers to and facilitators of the integration of complementary therapies in a hospital setting.

**Methods:**

We conducted a qualitative study with a descriptive approach by using focus groups with nurses working in a centre for integrative pediatrics in Switzerland. Focus groups, with overall 9 participants, took place in August and September 2023. The verbatim transcripts of the focus groups were thematically analysed with the assistance of a qualitative data analysis software. The Consolidated criteria for Reporting Qualitative research (COREQ) checklist was used to report the results of this study.

**Results:**

Three major themes were derived from data analysis: (1) influence of IM on the nurses’ job satisfaction and practice, (2) barriers to the practice of IM and (3) facilitators of the practice of IM. Nurses described multiple positive aspects of IM and how its practice influenced their satisfaction and motivation for their job. These aspects included more diversity in therapeutic options, greater autonomy and responsibility in therapeutic decision-making, and increased collaboration with parents. Barriers to the practice included workload, lack of knowledge about IM and complementary therapies, and inadequate billing processes. Among the facilitators were the presence of leaders to guide nurses in their practice of IM, in-house training and adaptation of IM protocols to the specific contexts of each unit in the department.

**Conclusions:**

The integration of complementary therapies and the transition from conventional care to IM seem to offer opportunities to better respond to the needs of nurses. However, despite the value that IM adds to nursing practice and the global satisfaction that it brings to nurses, some factors, mostly organisational and financial, challenge the wider recognition and integration of complementary therapies into practice.

**Supplementary Information:**

The online version contains supplementary material available at https://doi.org/10.1186/s12906-026-05533-w.

## Background

Nursing is a critical factor for the quality of care in hospitals. Because of their close and frequent interactions with patients and their central role in the delivery of care and treatments, nurses are key players of the interprofessional healthcare team. This important role applies in particular to the context of integrative medicine (IM) [1–3], which is increasingly being implemented in hospital settings [4, 5]. IM describes a patient-centred, relation-based and resource-orientated interprofessional approach that uses all appropriate therapeutic, preventive and health-promoting methods from conventional and complementary medicine (CM) to support optimal health, healing and development [6, 7]. Regarding nursing, IM is “a framework for providing whole person/whole system care, which is relationship-based and person-centred and focuses on improving the health and wellbeing of caregivers as well as those they serve” [8, p.2].

The health and well-being of nurses is of particular interest in the context of the current crisis of the nursing workforce, characterised by an increasing shortage of nurses and high turnover rates in the profession [9–11]. Burnout is a significant problem among nurses and is related to the special conditions of the nursing profession [12–16]. Further reasons why nurses leave their profession include disappointment in the nursing reality, the discrepancy between motivation/education and practice, a culture of hierarchy and discrimination, and poor working conditions and a challenging work environment [17]. Considering some observations in clinical settings, experts in the field of integrative nursing have suggested that integrative nursing can be a way to address some of these issues, notably regarding the discrepancy between motivation/education and practice, by focusing on whole-person, individualized, relationship-based, and meaningful care [8, 18, 19]. In addition, nurses have generally a positive attitude towards complementary therapies and, when the context favours the practice of IM, nurses are more satisfied with their job [20]. This can be explained by the fact that integrative nursing emphasizes principles and factors related to nurses’ job satisfaction, such as greater autonomy and decision-making, structural empowerment, self-growth and professional development [21, 22].

In pediatrics, IM is an emerging field that affects all areas from primary care to highly specialised medicine [23–25]. Introducing complementary therapies into conventional care requires close examination of the specific needs of the units or services in which they are meant to be implemented. The type of therapies introduced and how they are operationalized in practice depend on the intended goals of the integration program, on the specific institutional missions and resources as well as on the constraints imposed by the healthcare system in which they are implemented [3]. Integrated complementary therapies include pharmacologic and non-pharmacologic interventions and range for example from herbal remedies, aromatherapy, and acupuncture to hypnosis, mind-body techniques and external nursing applications [26–29]. Studies evaluating the transition from conventional medicine to IM in different pediatric inpatient settings have revealed the importance of information, knowledge, training and interprofessional collaboration during the transition [3, 26, 27, 30]. In addition, staff attitude and workload need to be addressed when developing a transition plan [3, 31]. However, little is known about the influence of IM on the nursing profession, or about the barriers to and facilitators of the practice of IM in daily work. To that end, it is necessary to study the experience of those directly involved in patient care and in the provision of complementary therapies in an IM setting.

In Switzerland in 2015, the Department of Pediatrics of the Fribourg Cantonal Hospital was the first public pediatric inpatient service to offer IM in its entire department. Following a successful pilot phase where protocols for complementary therapies were developed for specific conditions, the project expanded to become the Center for Integrative Pediatrics [28, 32]. From the outset, a strong emphasis was placed on strengthening the role of nurses. Indeed, nurses in the department were and still are actively involved in the creation and ongoing adjustment of the treatment protocols and provide most of the complementary therapies. However, since the pilot phase, which included a short survey on the satisfaction of the nursing staff [28], there has been no assessment of the impact of IM on nurses’ practice. The aim of this study was thus to explore nurses’ perceptions and experiences of practising IM in a pediatric hospital, as well as to identify barriers to and facilitators of the integration of complementary therapies in a hospital setting.

## Methods

### Study design

A qualitative study with a descriptive approach [33] was conducted through focus groups (FGs) that consisted of nurses working in the Center for Integrative Pediatrics of the Fribourg Cantonal Hospital. A descriptive approach was chosen as it allows to stay close to the data, while providing a rich and straight description of participants’ experiences [33]. A thematic analysis of the results was performed on the FGs transcripts [34].

### Setting

The Center for Integrative Pediatrics of the Fribourg Cantonal Hospital was established progressively since 2015 to meet the growing demand of the population for CM and, at the same time, to respond to the strong interest expressed by the medical and nursing staff in expanding the range of inpatient treatments offered [28, 32]. The center is divided into three units, each with their own organisation, chief nurse and staff: pediatric emergency, neonatology and inpatient pediatrics. The IM approach is implemented in all three units, with protocols adapted to the respective conditions of care. Complementary therapies that are essentially from anthroposophic medicine are offered in addition to conventional standard care following detailed treatment protocols. Anthroposophic medicine is an integrative medical system that provides a large and multimodal spectrum of complementary therapies [35, 36]. In Switzerland, it is integrated in the healthcare system and reimbursed in the framework of mandatory health insurance under certain conditions [37, 38]. In an inpatient setting, at least 10 CM treatment sessions have to be provided during the patient’s hospital stay before these sessions can be billed [38].

The Center for Integrative Pediatrics is co-directed by a nurse and a pediatrician, both specialised in anthroposophic medicine. Nurses play a central role in the delivery of complementary therapies, as they can initiate or suggest treatments autonomously by following the established protocols. These therapies include anthroposophic remedies and specific nursing applications such as wraps, compresses and embrocations. As an example, the CM treatment protocol for patients with respiratory diseases has been described in detail in a previous publication [28]. Both nurses and physicians receive a basic introduction to IM and anthroposophic medicine at the beginning of their employment. Nurses also receive practical training in external applications and are supervised by experienced colleagues in the workplace. All members of the interprofessional team are offered regular continuing education on various clinically relevant topics related to IM.

### Recruitment

Recruitment was conducted using convenience and purposive sampling techniques. To participate in the study, nurses had to meet two inclusion criteria: (1) to have been working at the Center for Integrative Pediatrics for at least 6 months and (2) to be willing to participate in a single FG for approximately 2 h in French. The only exclusion criterion was to be a chief nurse in one of the three units, in order to avoid hierarchy interference during the FGs.

Before recruitment took place, AR (Master’s thesis student in medicine) and BH (pediatrician and co-director of the Center for Integrative Pediatrics) met the chief nurses of the three units to explain the study and agree on a recruitment method. The chief nurses then sent an e-mail, together with an information sheet, inviting all nurses in their respective units to take part in FGs. Interested nurses could register by choosing from three proposed dates. To increase the recruitment rate, AR and BH were invited to present the study during staff turnover in the units. In addition, an information sheet containing a QR code to register in the study was left in each unit. Nurses were also informed that the time spent in the FG would count as working hours. After a first date was set, the nurses who could participate in the FG were contacted in order to give them more information about the interview’s location and time. Another wave of recruitment then took place, with the same procedure as occurred the first time, in order to carry out a second FG.

### Data collection

An interview guide was elaborated based on the study objectives, discussions within the team and the expertise of BH (pediatrician and co-director of the Center for Integrative Pediatrics). It was used to conduct all FGs (see Additional file 1). Questions revolved around nurses’ experience of practicing IM in the center, influence of IM on nurses’ perceptions of their profession, impact of IM on nurses’ work conditions, and influence of IM on interprofessional relations. The interview guide was not pilot tested. FGs took place in August and September 2023 in a quiet room of the hospital outside the pediatrics department. The FGs were moderated by JD (senior qualitative researcher), who animated and managed the discussion. AR was trained by JD to co-animate the FGs and helped with organisation, taking note of speaking turns and managing the recorder. The interviewers introduced themselves prior to the FGs and stated the study objectives. The information contained in the information letter was repeated, notably regarding data confidentiality, and room was given to answer any questions participants may have about the conduct of the study. Given the presence of the co-director of the center in the study team (but not during the FGs), reassurance was given to participating nurses that their identity would remain confidential and that they should feel free to voice their opinions without fearing consequences. All participants gave their oral consent to participate. No field notes were taken during the FGs. The FGs were audio-recorded and transcribed verbatim by AR. The transcripts were not returned to the participants.

### Data analysis

A thematic analysis [34] was performed with the assistance of the qualitative data analysis software MAXQDA 2022 (v. 22.8.0). As a first step, JD and AR read the transcripts to familiarise themselves with the data. The first transcript was then coded by two researchers (AR and JD) and compared to ensure consistency and agreement on the coding method. AR then coded the second transcript. Analysis was both deductive and inductive. Indeed, while our coding was primarily aimed at identifying how IM influences nurses’ practice, as well as the barriers to and facilitators of the practice, inductive coding was also performed in order to identify other aspects related to the practice of IM. As the analysis went on, the codebook was adapted iteratively when new codes were identified in the data. These changes were discussed between AR an JD. To ensure consistency in the coding process, AR wrote memos to describe each code and its meaning. All coded transcripts were cross-checked by JD to ensure the validity of the coding. After the codes were compared and similar codes merged, codes were classified into larger themes and subthemes. All transcripts were reviewed to identify the relationships between different themes within each FG and across FGs. Meetings were held with the co-authors (BH and PYR, physician and professor in primary care) to discuss the codes and interpretation of the data.

Our data are based on quotations of the participants. AR translated the quotations that are used in the manuscript from French to English, with the help of JD. An additional reading was then performed by a native English speaker to make sure the idiomatic meaning of the phrases of the FG participants was preserved. Parentheses in the quotations indicate omission of utterances in order to create cohesive sentences. We used the Consolidated criteria for Reporting Qualitative research (COREQ) checklist to report our study [39].

### Reflexivity statement

This manuscript is the result of AR’s (first co-author) Master’s thesis. While looking for a research topic for her thesis and considering a future career in pediatrics, AR approached BH (pediatrician and co-director of the Center for Integrative Pediatrics) and PYR (physician and professor in primary care), who proposed the topic and became her Master’s thesis supervisors. Given the qualitative orientation of the study, JD (anthropologist and senior qualitative researcher specialized in primary care and complementary medicine research) joined the team to support the design and conduct of a qualitative study using a descriptive approach and to provide methodological guidance throughout the research process.

Given BH’s position as co-director of the Center for Integrative Pediatrics from which participants were recruited, it was decided that he would not participate in data collection and would not have access to transcripts before they were deidentified. These measures aimed to reduce the risk of identification and minimize potential hierarchical influence during data collection.

Although the study adopted a descriptive and minimally interpretive approach, no research process is free from the perspectives and assumptions of the researchers involved. Throughout the analysis, AR kept analytic memos documenting coding decisions and reflections. These were discussed with JD during the iterative refinement of the coding framework. Furthermore, discussions among co-authors with different professional and methodological backgrounds allowed different perspectives on the data to be considered and contributed to strengthening the credibility of the analysis.

## Results

Nine nurses (9 of 87; 10%) participated in the two FGs. The 78 nurses who did not participate either were not available at the interview dates, or did not wish to participate for undisclosed reasons. The FGs lasted 1 h 37 min and 1 h 47 min. Five nurses worked in neonatology (5 of 25; 20%), one in inpatient pediatrics (1 of 26; 4%) and three in pediatric emergency (3 of 36; 8%). All but one of the nurses were already working in the department of pediatrics before the transition from conventional care to IM. Some nurses have changed units within the pediatric department since they started working there.

The analysis of the FGs resulted in three main themes: (1) influence of IM on the nurses’ job satisfaction and practice, (2) barriers to the practice of IM and (3) facilitators of the practice of IM. The coding tree resulting from our analysis is provided as an additional file (see Additional file 2).

### Influence of IM on the nurses’ job satisfaction and practice

Participants described elements that motivated them to practise IM and how the experience of IM influenced their general satisfaction.

#### Perceived positive aspects of IM on nursing practice

Multiple positive aspects of practising IM were perceived by nurses. Notably, practising IM helped some nurses gain confidence in their everyday practice and in their patients.

“What I have learnt is to trust the [patient’s] body, too. Before, I was perhaps a younger graduate, when there wasn’t yet anthroposophic medicine, but I didn’t like a baby with a fever, (…), I was more quickly stressed. And with the contribution of these [complementary therapies], I’ve learnt to be more trusting, but I’ve also developed my nursing and personal practice (…).” (FG2, nurse 4, emergency)

IM taught nurses to observe patients more and to base their practice on observation rather than on numbers from the monitor, especially in the neonatology unit.

“I’d say that what was incredible in terms of my observation of the staff was all this clinical perception that we were able to develop as nurses. We were very attached to the monitor, (…), at least in our case, in neonatology, and I find that there’s a kind of trust, there’s something that’s been created in the invisible, I would say.” (FG1, nurse 2, neonatology)

Participants also underlined autonomy in the delivery of complementary therapies as a positive aspect. Indeed, although they must follow protocols, nurses can start some CM treatments before the approval of a physician, if the therapeutic indications are right.

“What’s really interesting is that as a nurse [practising IM], we still have certain freedoms to feel things, suggest this or that therapy, (…). There are little things that we can also feel, that maybe it would be good to try putting this substance on.” (FG1, nurse 3, neonatology)

Another benefit perceived by almost all nurses was that complementary therapies provided them with an additional toolbox to offer to patients, notably in cases where conventional medicine seemed limited.

“With these bronchiolitis cases, before, when we weren’t doing [IM], apart from feeding them and cleaning up everything they had thrown up, we weren’t doing much to care for them. (…) There is no treatment, so you just have to wait it out until they [the patients] reach the lowest point and then they will start to get better. But IM is a great support for them.

For these very problems, I’m much more satisfied now that we are using anthroposophical medicine.” (FG2, nurses 3 and 5, neonatology)

Finally, the possibility of parents providing certain complementary therapies was also perceived as a benefit from IM.

“It leaves room for the family to participate in the care. In the end, we know very well that without the patient’s parents, we wouldn’t be able to work. And without their parents, patients would be in even worse shape. So, I find that sharing knowledge and responsibility is all the more important for the parents and the relationship with us. It’s the three parties: the child, the parents, the nurses.” (FG1, nurse 4, emergency)

#### Motivation

Nurses highlighted different elements that were considered important to stay motivated to practise IM. Most nurses underlined that being able to witness the effects of complementary therapies on children was both a source of satisfaction and an important motivator for practising IM. Nurses further explained that the patient’s length of stay was determinant in witnessing an effect. Indeed, although children in neonatology and in inpatient pediatrics would stay for a few days or weeks, children in the emergency unit stayed a maximum of 6 h, which made it more difficult for nurses to see an effect. When nurses could not see the effect of the CM treatment, they described feeling frustrated and were less likely to continue practising that care.

“You [in neonatology], you see this effect, it’s much more stimulating, and it’s much more gratifying and it should make you want to carry on. In the [emergency unit] it’s just a workload, but we don’t see the positive side of it, and there’s nothing worse than that. If you give an antibiotic for periorbital cellulitis, the next day [the child will] open his eye. There you go. Unfortunately, there’s probably an effect [of CM], but you never see it. And I think that’s very frustrating when you actually do it. You get the impression that you’re of no use with it.” (FG2, nurse 2, emergency)

Furthermore, motivation to practise IM came through experience, as this nurse stated, based on the observation of her colleagues:

“For example, if I take our team, there really were some extremely resistant people from the start. And these people, now, without a thousand speeches, a thousand theories, a thousand training courses, a thousand whatever, I find they’ve really changed in their approach.” (FG1, nurse 2, neonatology)

When convinced, nurses seemed to be more motivated and more invested, even if it meant working overtime or missing breaks.

“We know that it will improve the child’s well-being because we’re really convinced of that. So we have to do what we have to do (…). Because it [the IM approach] reduces the time spent in hospital for bronchiolitis, for example, with body wraps and all the other things we do, so it’s huge! And just for that, it’s worth it anyway, even if it means we’ll have two minutes less of a break.” (FG1, nurse 4, emergency)

For two nurses, working with IM became the reason they continued working at the hospital. As one stated:

“In any case, (…) this integrative medicine is what keeps me working in the hospital. (…) I don’t think I can put it any other way, (…) and I now feel complete in my profession. For years, I loved my job deeply for all the right reasons, but I felt that something was missing, to be quite honest. And when I started all this complementary medicine, I felt that there were bits that were coming to light.” (FG1, nurse 2 neonatology)

#### Adoption of CM as routine care

Some of the participants’ statements unveiled elements indicating the extent to which CM was now integrated into conventional care and adopted as routine care, thus suggesting a transformation of nurses’ practice. In neonatology, nurses shared that two-thirds of the medications routinely used in the unit were now medicinal products from anthroposophic medicine. For some conditions, they almost exclusively used CM.

“For example, we use [CM eye] drops against conjunctivitis. And it’s true, since we’ve been using these, we hardly ever use antibiotic eye drops any more. (…). I once went to pick up a baby in [another hospital] for a transfer. Then they said, “He’s still being treated with Floxal drops [antibiotics]”. I was like “Oh yes, those still exist.” (FG2, nurse 5, neonatology)

Still, in neonatology, nurses of one FG shared their concerns about a randomised controlled trial that would be taking place a few months later. They felt worried that they would no longer be authorised to give anthroposophic care to the patients randomised as such.

“And then, emotionally, it’s going to be a problem for me because those [patients] who don’t have anthroposophic medicine, I have the feeling that I won’t be doing my job properly.” (FG2, nurse 3, neonatology)

Nurses also narrated examples of how they self-appropriated IM to integrate it into their practice, as one nurse states below.

“Clearly, I use lavender everywhere. Because even for babies in [neonatal drug] withdrawal, a lavender bath at 8 o’clock in the morning is the sign of a good day.” (FG2, nurse 1, neonatology)

### Barriers to the practice of IM

#### Workload

Several nurses underlined that they initially feared that the transition from conventional care to IM, with the introduction of complementary therapies, would bring an extra workload.

“(…) at the very beginning, when we started using it [CM], when we talked about it, maybe it was a bit scary, we thought maybe it was an extra workload, something else again.” (FG1, nurse 3, neonatology)

Most nurses from the emergency unit considered it later to be proved true, and that the workload, a stressful context and understaffing prevented them from practising IM.

“It really weighed on me when I found out that we were also starting [with complementary therapies] in the emergency unit. And to be honest, I don’t do it very often, if ever. (…) Because of the workload. Because there are a lot of things in conventional care that we don’t have time to do, that we do badly because we don’t have time. (…). And that [CM], for me, was really an extra burden.” (FG2, nurse 2, emergency)

Although most of the other participants also acknowledged an increased workload at some point, they did not necessarily consider it a barrier to practise IM, for two reasons. First, nurses stated that, with experience, CM therapies became easier and faster to perform.

“It’s a routine, too, because at the beginning, or even now, if I haven’t done it [CM therapy] for some time, for a body wrap, I’ll go and look in the binder: what I’m preparing, how much ginger I’m going to take, how much water, all these things that if you do it regularly, it’s in your head, you don’t waste time getting out the binder and checking it again.” (FG1, nurse 1, inpatient pediatrics)

Second, although the work required to perform complementary therapies and adhere to the protocol was indeed considerable, it ultimately saved them more time than it took to implement when the duration of the whole hospital stay was taken into account.

“And what I’d like to add about this time, is that it takes time at the beginning, but it’s true that if you take the whole period of hospitalisation, everything balances out. So yes, it takes time at first, twice, even three times [more time], but if you take everything together, I don’t think it takes any longer. In any case, it’s something I’ve often thought to myself about the fact that the child is calmer, that the parents are more reassured, they ring the bell less.” (FG1, nurse 2, neonatology)

Finally, nurses from the neonatology unit, where patients stay longer than in the emergency and pediatrics units, considered the involvement of parents in the care of their child to be a relief, especially when the workload was very high. Indeed, in this unit, parents often learn how to do some embrocations, massages or inhalations for their children.

“Yes, but when you’ve got a heavy workload, you’re happy that the patient’s dad is the one to handle the spray cans or whatever.” (FG2, nurses 3, neonatology)

#### Discipline specific factors

Some of the barriers mentioned by the participants were related to the specific context of pediatrics. First, several nurses identified the patients’ age as having an impact on the feasibility of delivering IM. Some therapies, such as body wraps, were considered easier to perform on younger children. In contrast, older children were sometimes reluctant to have any kind of therapy, which made the entire care more challenging.

Second, seasonal diseases, especially bronchiolitis, were affecting all units negatively. The resulting higher number of patients and heavier workload often led nurses to leave some complementary therapies aside.

“When we have a heavy workload, like this epidemic last winter, we do what’s quick and easy to do, especially for the night, it’s the lavender, the other wraps; during the day, it’s often put aside.” (FG1, nurse 1, pediatrics)

#### Technical barriers

Some barriers were based on technical challenges, such as mastering different substances, or not having adequate space or material to practise. For example, in the emergency unit, nurses did not have access to a fridge, which was needed to store some ingredients for complementary therapies, and they did not have the space to prepare the substances outside of the patient’s room. In neonatology, a different layout of the nursing ward helped improve staff dynamics and relationships with the parents.

#### Lack of knowledge about IM

Three nurses explained that IM and complementary therapies sometimes went against what they had learnt at nursing school: for example, they were bothered by the fact that quantities did not depend on the patient’s weight, as is the case in conventional medicine.

“We use [potentised] stibium [complementary therapy], and whatever the child’s weight, we always give the same [number of] drops (…). Whether he’s 6 months old or 1 month old. (…) And one day, at the visit, I told the doctor that this didn’t suit me. Everything is based on weight, and now you’re telling me I have to give 5 drops. (…). Well, I have to admit that sometimes I ‘forget’ to give stibium if I don’t have the time.” (FG2, nurse 1, neonatology)

Two of these three nurses shared that they also felt wrong giving out a preparation without knowing the exact effects and possible side effects of that therapy and felt uneasy explaining to the parents something they did not really master.

“And personally, I found it stressful to go towards parents with substances that I didn’t really know much about and that… In terms of effectiveness, I wasn’t that convinced either. So, it’s true that in the beginning, I remember that I was part of the reluctant people” (FG2, nurse 3, neonatology)

#### Billing process and impact on the practice

The billing process had an impact on the nurses’ sense of recognition of their practise of IM. Indeed, in the current Swiss inpatient case-based reimbursement system, at least 10 CM treatment sessions have to be provided during the patient’s hospital stay before these sessions can be billed. When the hospital stay was short, it was difficult to get to 10 sessions and thus be billed, which did not motivate some nurses to continue practising IM.

“And, above all, it’s not prescribed, it’s not… nobody checks, nobody cares, (…). What’s more, it’s not paid, it’s not recognised, (…). And finally, it’s true that your actions aren’t really recognised, that what you do takes up a lot of your time and isn’t billed a penny.” (FG2, nurse 4, emergency)

### Facilitators of the practice of IM

#### Presence of IM leaders

The two co-directors of the Center for Integrative Pediatrics were working in neonatology. Working with them was considered by all participating nurses from this unit to be an advantage because they were able to guide nurses in their practice of IM.

“It’s a very, very positive effect, because clearly, every time we have a little question, we always have more or less the opportunity to ask. (…) It’s true, we’re very lucky that she [nurse specialised in anthroposophic medicine] is there, she always answers with great pleasure, and then the fact of collaborating with [pediatrician specialised in anthroposophic medicine] who is very present, then it’s all this exchange of our observations, and then we’re involved, we’re really a team.” (FG1, nurse 3, neonatology)

The presence of these leaders also seemed to influence communication within the unit.

“…or even at the level of transmissions, of relationships, there’s a kind of dimension that’s been put in place without us being able to name it, let’s put it that way, which is a bit specific, I think, to complementary medicine. And, yes, it has brought a great deal to the table. And also, in terms of collaboration with doctors, there’s also something that’s really evolved in a very pleasant way. Before, it was more like everyone working in their own corner, but now we’re together with the patient, with the parents, there’s a real dimension that has, for me at least, grown over the years” (FG1, nurse 2, neonatology)

Yet, nurses from other units did not describe such an effect on group dynamics and interprofessional collaboration: in their units, collaboration with pediatricians regarding IM was not as present, possibly because of the lack of time from both nurses and doctors, as well as pediatricians’ lack of knowledge about IM.

#### Context of care

Nurses of the second FG compared the three units and the possibility of planning the provision of complementary therapies ahead of time. In neonatology, nurses were able to plan their day around IM, allowing them to follow protocols and patients’ needs. Most nurses from this unit stated that by the end of their shift, they mainly managed to perform all the therapies planned, which was not the case in the emergency unit.

“As someone who has moved from the inpatient pediatrics unit to the emergency unit, the big difference in the practice of anthroposophic medicine is that when you have two or three children, you can plan your day in the morning and say to yourself, I’ll do it between 9 and 10 because I have less workload. I’ll maybe put it between 15 and 16, we’ll put it back in the evening, (…), you can still plan a time during the day (…). And then, on the other hand, in emergency, you can’t anticipate anything, so they [patients] arrive, you can’t say to yourself, I’ll do it in an hour, I’ll do it when I’ve got the time, you can’t.” (FG2, nurse 2, emergency)

To be able to practise IM, some nurses believed it had to be done in a quiet environment, when the patient and the nurse were calm. For one nurse, it was not worth it to try practising IM without this quiet environment:

“For me, it’s part of the concept. It’s part of the treatment, I’d say. You have to do it when you’re in the right conditions, and if you can’t do it in the right conditions, you might as well not do it. And I think I’ve understood that.” (FG2, nurse 2, emergency)

Two nurses, in contrast, disagreed with that affirmation. One of them thought that some nurses used those unmet conditions as an excuse to practise IM less.

“So with us [in inpatient pediatrics] in terms of reluctance, what I often hear is, “The child wasn’t well enough, so I didn’t start the wraps, I didn’t do the aerosol treatment, there was the high-flow (…), I didn’t want to do the [CM] aerosol over it yet”, or things like that, that I still hear regularly, that’s a shame.” (FG1, nurse 1, inpatient pediatrics)

#### In-house management

Nurses identified facilitators of the practice of IM that were related to institutional decisions, such as having adequate training and adaptable protocols. First, seven nurses underlined the need to be trained in IM before working in the center. In fact, all seven of them found that having at least a one-day training in IM and complementary therapies was necessary to understand the basics.

“I think it’s important [to get trained]. To remain consistent because it’s important to know what you’re treating and to understand the whole philosophy behind it. (…) It’s got nothing to do with what we’re taught in our nurse curriculum.” (FG2, nurse 4, emergency)

One of the nurses started working in the center one and a half years before the FG took place and never participated in training. She stated that knowing more about IM would encourage her to practise.

Second, the center adapted protocols on complementary therapies and the IM approach, depending on the units. For example, the spectrum of CM therapies offered in the emergency unit was reduced because nurses lacked time to practise some therapies such as curd wraps or specific CM aerosol therapies. They now only have the other wraps available, which are easier to make and take less time. Furthermore, having fewer different substances and protocols to master, as was also the case in neonatology, made IM more approachable for nurses. Five nurses across units talked about thyme wraps, which were quite complicated at first, especially for beginners. Now, they have beeswax sheets with thyme that are ready to apply, which facilitates care and gains time. This seemed to be very appreciated by nurses.

“After 20 minutes or half an hour, we’d have to take it [body wraps] off and check the temperature, because it’s damp and babies get cold very quickly. We had to wake the child up again to take it off. Now it’s much simpler. They also realised that it wasn’t possible in real life. At least not in the hospital.” (FG2, nurse 5, neonatology)

## Discussion

The influence of IM on everyday practice, as well as barriers to and facilitators of the practice of IM, were made visible through this qualitative study conducted in the Center for Integrative Pediatrics in Switzerland. Overall, our findings suggest that IM can create added value to the nursing profession in a hospital department.

Nurses described multiple positive aspects and advantages of IM and how the practice of IM influenced their satisfaction and motivation for their job. This can be linked to factors such as a more patient-oriented and less technically focused approach, having more therapeutic options (a greater toolbox) for patient care through the integration of complementary therapies, having greater autonomy and responsibility in therapeutic decision-making, as well as increased collaboration with parents as the primary caregivers of their pediatric patients. In addition, the nurses expressed an increase in self-confidence towards their clinical practice and in the appreciation of their own work following the transition from conventional care to IM. They also reported that they had developed greater confidence in the patient’s natural regulatory and self-healing capacities, which are supported by the CM therapies. Finally, some nurses identified IM as the main reason for not leaving their workplace in the hospital.

These findings are in line with those from previous studies in different settings. Like other healthcare professionals, nurses generally appreciate the availability of an extended range of treatments offered by IM [1, 20, 26, 30, 40]. From a systematic review of studies investigating nurses’ attitudes towards complementary therapies, Hall et al. [20] describe the greater job satisfaction of nurses through the practice of IM and the available additional therapies from CM. By highlighting the boundary role of nurses in the delivery of healthcare in hospitals, Arnon et al. [1] found that the gain in personal knowledge, self-empowerment and the range of CM treatments adapted to patient needs favoured a positive attitude, satisfaction and motivation among nurses. The more patient-centred approach of IM also encourages and requires closer contact with patients and their families. This was particularly appreciated by both nurses and parents in a study in pediatric oncology [26]. The close contact with patients also helps nurses to better identify patients’ needs and detect situations in which complementary therapies might be helpful [1, 30]. In addition, in our study, nurses’ autonomy in therapeutic decisions based on predefined treatment protocols enhanced satisfaction. The active involvement of nurses in developing the IM treatment protocols, as was the case in the Center for Integrative Pediatrics, might have further contributed to the overall satisfaction of nurses. Studies have shown that enhanced professional autonomy may have a positive influence on job satisfaction and may thus contribute to promoting the retention of nurses in the profession [41, 42]. Conversely, lack of professional autonomy and the institution’s reticence to allow IM have been described as barriers to its integration [20] and are a potential reason for frustration and dissatisfaction among nurses.

As in all areas of healthcare, an important aspect for caregivers is the possibility to observe the effects of therapies. Seeing the benefits for patients also enhances job satisfaction and interest in IM [3, 30, 43]. The nurses in our study highlighted this aspect as promoting their personal motivation to practise IM. If the setting does not enable the observation of effects, or when treatment effects are not immediately apparent, as with many therapies from CM, it is more difficult to develop this conviction or to be motivated to deliver those therapies. The mostly short hospital stays for acute illnesses in children, as well as billing systems that favour early discharge, are disadvantageous in this context. Asking parents for feedback about treatments even after discharge might be helpful for continuous evaluation and development.

Our study identified several barriers to the practice of IM in a hospital setting. First, nurses mentioned workload, time constraints and understaffing as major barriers for the implementation of additional CM therapies. Seasonal variation of diseases, as well as stressful and unpredictable situations such as in an emergency department, contribute to the challenges of the workload. The possible impact of additional work due to complementary therapies was also the main concern of the nurses before IM was introduced in our hospital. As shown by other investigators, staff attitude and workload must be taken into account in the implementation of IM [3, 31]. Previous research already demonstrated that a heavy workload and lack of time resulted in nurses practising less CM [2, 3, 20] and being more frustrated [30]. Beyond workload, understaffing creates a resistance to all types of change in nursing practice, including IM [26].

Yet, workload was not always considered a barrier to the practice of IM, as previously described [44]. During longer hospital stays, nurses emphasised that the extra workload invested in the beginning was levelling out during hospitalisation. In addition, involving parents in providing some of the complementary therapies also relieved nurses’ workload and contributed to mutual satisfaction. This opportunity to involve parents as caregivers has been reported in other studies [2, 30] and could be a general path for optimising patient care.

Lack of knowledge about IM and complementary therapies is a major barrier for the implementation and daily practice of IM. All nurses in our study agreed that basic training is needed at the beginning of working at the center, and regular training should be provided to improve IM knowledge and the delivery of complementary therapies. Staff education and support are key elements to foster the transition of care and should receive sufficient resources [2, 3]. Considering CM and IM during pre-graduate training in nursing school curricula could be a way to better inform and prepare nurses for the practice of IM.

An unexpected finding in our study was that the billing process of complementary therapies had an impact on the nurses’ feeling of recognition. In fact, when therapies could not be billed, nurses were less motivated to deliver them. Although Switzerland has a reimbursement policy for complementary therapies delivered in inpatient contexts, it is not yet adequately adapted for acute settings or complex cases [38]. In addition to impeding the valorisation of these therapies, this situation further limits the availability of the necessary time and personnel to perform them. Inadequate or non-existent reimbursement has also been identified by other investigators as a barrier to the use of CM in clinical practice [45, 46].

Finally, our study identified various facilitators of the practice of IM. Positive group dynamics with IM leaders who share knowledge and provide supervision was discussed as a stimulating factor. This confirms previous observations that have pointed to the importance of leaders for teaching, visibility and sustainability of IM projects [3, 31]. Training professionals to become IM leaders could be a first step to better support nurses in practising IM. Although our findings regarding interprofessional collaboration are limited, we could show that the implementation of CM therapies and the transition to an IM approach does not automatically improve or foster interprofessional collaboration. As the collaboration of different healthcare providers is a key element of the multiprofessional approach of IM, our findings confirm the need to take this separately into consideration during the transition process [3, 26, 27, 30].

Having enough space for preparing and delivering the CM therapies, availability of materials, and planning of patient care are other facilitators identified in our study. The technical and organisational conditions are context specific. This highlights the need to adapt the IM approach to the specific culture and conditions of a unit or department. Finally, it is evident in the discussion with the nursing staff that satisfaction and motivation are themselves facilitators of the daily practice of IM.

From a nursing theories perspective, our findings might be discussed and further explored under the frameworks of person-centred nursing [47, 48] and family-centred pediatric nursing care [49]. As demonstrated by our study, nurses’ commitment to their job, a prerequisite for person-centred nursing [47], can be fostered by the transition from conventional care to IM through increased motivation and satisfaction. IM has an impact on the culture of the workplace and the care environment. Further, the integration of CM therapies as innovative practices can strengthen the therapeutic role of nurses. Person-centeredness is also reflected by the experiences of nursing staff, who report that the IM approach leads to a stronger focus on patients with closer clinical monitoring, increased confidence in patients and their natural recovery processes, and the creation of relationships with therapeutic benefit. The relationship between families and healthcare professionals is also the core of the definition of family-centred care [49]. Our findings suggest that the integration of CM therapies can be a way to foster a collaborative partnership between nurses and families by the active involvement of parents in the treatment and care of their child. This has already been demonstrated in another study from our centre, where lavender oil chest wraps as CM therapy offered a sense of agency to both nurses and parents, and nurses emphasized that involving parents can make nursing procedures more reassuring for infants and, at the same time, empower parents in their caregiving role [50].

This study provides valuable insight into nurses’ everyday practice in a pediatric hospital department where IM is already implemented. To our knowledge, it is the first qualitative study on the subject in Switzerland. Unlike studies investigating the perceptions and experiences of nurses during or shortly after the implementation phase of complementary therapies, our study took place more than 8 years after the introduction of IM, which was associated with several years of experience and exposure among the participants. Furthermore, within one study, we were able to explore nurses’ experiences from three different units in the same department, underlining how different contexts of care may influence the practice of IM and shedding light on strengths and areas that require closer attention. Nonetheless, there are several limitations to this study. First, recruitment was challenging because of time and scheduling constraints, resulting in the inclusion of a limited number of participants. Consequently, the findings reflect the perspectives of the participating nurses and should not be assumed to represent those of the entire nursing staff at the center. Second, the representation of only one nurse from the pediatrics unit makes it difficult to draw conclusions about that unit. Further studies could focus on that unit and integrate other healthcare professionals. Third, there may have been a potential selection bias, as nurses willing to participate might have been more open to IM or may have had more positive experiences with IM, and because we did not account for nurses’ level of education regarding CM in our inclusion criteria. Furthermore, recruitment resulted in an overrepresentation of nurses working in the unit of the persons identified as leaders. Those elements may have skewed the results towards a more positive view of IM than is the case.

## Conclusion

Overall, our findings from the context of pediatrics suggest that nurses working in this IM setting perceived that IM contributed positively to their professional satisfaction. Thus, the integration of complementary therapies and the transition from conventional care to IM seem to offer opportunities to better respond to the needs of the nursing staff in hospital settings. However, despite the value that IM adds to nursing practice and the global satisfaction it brings to nurses, some factors, mostly organisational and financial, challenge wider recognition and integration of therapies from CM into practice. Adequate staffing, optimised organisation for an adapted workload and better integration of IM into healthcare professionals’ pre- and postgraduate education are key to supporting the integration of IM in inpatient care in a sustainable manner. This is important because IM, by bringing nurses greater satisfaction, as well as additional skills and autonomy, could be one way to address the problem of the nursing workforce crisis and to support a more sustainable healthcare system. Further studies should be conducted in different settings and at a larger scale including multicenter qualitative and/or quantitative studies involving hospitals at different stages of IM implementation to increase the understanding of the influence of IM on nurses’ practice, self-esteem and job satisfaction in order to guide policymakers and medical education leaders. This could include studies in hospitals from different healthcare systems and various medical specialties to distinguish between context-specific, discipline-specific and IM-specific aspects and determine whether the findings are transferable to other settings.

## Supplementary Information

Additional file 1. Interview guide.

Additional file 2. Coding tree.

## Data Availability

The interview transcripts are not publicly available, as consent for sharing data was not granted by the participants but are available from the corresponding author on reasonable request.

## Abbreviations

CM: Complementary medicine
FG: Focus group
IM: Integrative medicine
