# Supplementary material for: Added value of integrative medicine to the practice of nursing in hospitals: results from a qualitative study in pediatrics
Source: BMC Complement Med Ther. 2026 Aug 19;26:352. doi: 10.1186/s12906-026-05533-w (PMC13760895; doi:10.1186/s12906-026-05533-w)
Supplement: Supplementary file 1 [file 12906_2026_5533_MOESM1_ESM.docx]

| **Theme** | **Questions** | **Follow-up questions** |
| --- | --- | --- |
|  |  |  |
| **Introduction** |  |  |
| **Practising integrative medicine** | 1. What was your experience of integrative medicine before working at the center? 2. What has been your experience of integrative medicine since you started working at the center? 3. How much importance does integrative medicine have in your daily practice? 4. How does integrative medicine influence your actual practice? | -To what extent did you have an interest in complementary medicine before working in the center?  -Do you have additional training in complementary medicine (apart from the training received in the center)?  -Do you practise complementary medicine outside the hospital? |
|  |  |  |
| **Perception of the nursing profession** | 5. To what extent does the practice of integrative medicine influence your perception of your profession? 6. What impact does the integrative medicine approach have on your relationship with patients? | - To what extent does the practice of integrative medicine influence your motivation to practise nursing?  - What do you think is the most important thing in your job? |
|  |  |  |
| **Working conditions** | 7. To what extent does practising complementary therapies affect your workload? 8. To what extent does practising complementary therapies affect your job satisfaction? 9. What impact does the integrative medicine approach have on your relationship with the patients? | -What factors do you think are important for job satisfaction? |
|  |  |  |
| **Interprofessional relations** | 10. To what extent does the practice of integrative medicine influence interprofessional relations? |  |
|  |  |  |
| **Training** | 11. How much do you know about complementary therapies? 12. In your opinion, is basic training in complementary therapies necessary to work in the center? |  |
|  |  |  |
| **Conclusion** |  |  |
