# Supplementary material for: Added value of integrative medicine to the practice of nursing in hospitals: results from a qualitative study in pediatrics
Source: BMC Complement Med Ther. 2026 Aug 19;26:352. doi: 10.1186/s12906-026-05533-w (PMC13760895; doi:10.1186/s12906-026-05533-w)
Supplement: Supplementary file 2 [file 12906_2026_5533_MOESM2_ESM.docx]

| **Themes** | **Sub-themes** | **Codes** |
| --- | --- | --- |
| Influence of IM on the nurses’ job satisfaction and practice | Perceived positive aspects of IM on nursing practice | Gaining confidence |
|  |  | Greater autonomy |
|  |  | Additional toolbox |
|  |  | Inclusion of parents |
|  | Motivation | Witnessing effects |
|  |  | Motivation improved through experience |
|  |  | Influence of IM on intention to leave |
|  | Adoption of CM as routine care | Transformation of nurses’ practice |
|  |  | Self-appropriation of IM |
| Barriers to the practice of IM | Workload | Concerns prior to the transition |
|  |  | Special context of the emergency unit |
|  |  | Decrease with practice |
|  |  | Balanced overall |
|  |  | Involvement of the parents |
|  | Discipline specific factors | Patients’ age |
|  |  | Seasonal diseases |
|  | Technical barriers | Mastering different substances |
|  |  | Access to material |
|  |  | Architectural constraints |
|  | Lack of knowledge about IM | Incoherences regarding usual nursing practice |
|  |  | Uncertainty regarding (side) effects |
|  | Billing process and impact on the practice | Billing conditions |
|  |  | Lack of recognition |
| Facilitators of the practice of IM | Presence of IM leaders | Guide practice |
|  |  | Improved communication |
|  |  | Absence of leaders |
|  | Context of care | Ability to plan treatments |
|  |  | Quiet environment |
|  | In-house management | Training offers |
|  |  | Adaptation of protocols |
